# Application of Plastic Wastes in Construction Materials: A Review Using the Concept of Life-Cycle Assessment in the Context of Recent Research for Future Perspectives

**DOI:** 10.3390/ma14133549

**Published:** 2021-06-25

**Authors:** Tulane Rodrigues da Silva, Afonso Rangel Garcez de Azevedo, Daiane Cecchin, Markssuel Teixeira Marvila, Mugahed Amran, Roman Fediuk, Nikolai Vatin, Maria Karelina, Sergey Klyuev, Maciej Szelag

**Affiliations:** 1 Department of Agricultural and Environmental Engineering, UFF—Federal Fluminense University, Rua Passo da Pátria, 156, Niterói 24210-240, Brazil; tuhrodrigues_@hotmail.com (T.R.d.S.); daianececchin@id.uff.br (D.C.); 2 Advanced Materials Laboratory (LAMAV), UENF—State University of the Northern Rio de Janeiro, Av. Alberto Lamego, 2000, Campos dos Goytacazes, Rio de Janeiro 28013-602, Brazil; markssuel@hotmail.com; 3 Laboratory of Civil Engineering (LECIV), UENF—State University of the Northern Rio de Janeiro, Av. Alberto Lamego, 2000, Campos dos Goytacazes, Rio de Janeiro 28013-602, Brazil; 4 Department of Civil Engineering, College of Engineering, Prince Sattam Bin Abdulaziz University, Alkharj 16273, Saudi Arabia; m.amran@psau.edu.sa; 5 Department of Civil Engineering, Faculty of Engineering, Amran University and IT, Quhal 9677, Yemen; 6 Polytechnic Institute, Far Eastern Federal University, 690922 Vladivostok, Russia; 7 Peter the Great St. Petersburg Polytechnic University, 195251 St. Petersburg, Russia; vatin@mail.ru; 8 Moscow Automobile and Road Construction University, 125319 Moscow, Russia; karelinamu@mail.ru; 9 Department of Theoretical Mechanics and Strength of Materials, Belgorod State Technological University Named after V.G. Shukhov, 308012 Belgorod, Russia; klyuyev@yandex.ru; 10 Department of Building Construction, Lublin University of Technology, 20001 Lublin, Poland; maciej.szelag@pollub.pl

**Keywords:** lifecycle, circular economy, PET, construction, sustainability

## Abstract

The urbanization process contributes to the growth of solid waste generation and causes an increase in environmental impacts and failures in the management of solid waste. The number of dumps is a concern due to the limited implementation and safe disposal of this waste. The interest in sustainable techniques has been growing in relation to waste management, which is largely absorbed by the civil construction sector. This work aimed to review plastic waste, especially polyethylene terephthalate (PET), that can be incorporated with construction materials, such as concrete, mortars, asphalt mixtures, and paving. The use of life-cycle assessment (LCA) is related, as a tool that allows the sustainability of products and processes to be enhanced in the long term. After analyzing the recent literature, it was identified that studies related to plastic wastes in construction materials concentrate sustainability around the alternative destination of waste. Since the plastic waste from different production chains are obtained, it was possible to affirm the need for a broader assessment, such as the LCA, providing greater quantification of data making the alternative processes and products more sustainable. The study contributes to enhance sustainability in alternative building materials through LCA.

## 1. Introduction

The global urbanization process is one of the main factors responsible for the substantial growth in the generation of solid wastes, demanding attention for the increase in environmental impacts caused by the accumulation and failures in the management of solid wastes. Developing countries do not have a standard with regard to waste management practices and this fact contributes to the application of informal process models and, consequently, favors irregular disposal, such as in open dumps [1]. The lack of dumps represents a challenging factor due to the limited areas available for the implantation of sites for disposal and contamination risks [2]. The generation of solid waste is increasing in China, in addition to other parts of East Asia and in regions of Eastern Europe and the Middle East [3].

Currently, the world generates 2.01 billion tons of solid urban waste per year, at least 33% of which are not managed in an environmentally safe manner, in addition to a growth forecast of 3.40 billion tons by 2050 [4]. Underdeveloped countries and developing countries face a lack of infrastructure and adequate processing of waste. In developed countries, on the other hand, it is possible to relate the misuse of resources [5]. Sustainable solid waste management has become a necessity for industries seeking to promote industrialization and sustainable development [6]. Japan, for example, uses waste management, where although almost 44 million tons of waste are generated annually, only 1% is disposed in dumps, with the rest being recycled or converted into energy in state-of-the-art facilities [4]. 

Countries, such as Nigeria, Bangladesh, Sudan, and Ethiopia, are the main contributors to the production of solid waste among underdeveloped countries [5]. Senegal, for example, produces more than 2.4 million tons of waste per year. However, about 1.08 million tons are not collected [4]. According to [6], the recent development with respect to environmental policies has led to several sustainable approaches in relation to integrated solid waste management. In Brazil, the national solid waste policy was established by law 12.305/2010 and regulated by Decree 7.404 of 23 December 2010 [7]. Oliveira et al. [8] emphasizes that strict government regulations around the world have become a factor in accelerating the adoption of reverse logistics initiatives, thus wastes have gained a new meaning within their life cycle [9].

It is estimated that 6.2% of Brazil’s gross domestic product (GDP) is represented by the civil construction market [10]. The construction industry is one of the main sectors that have the potential to reuse solid waste, as a potential raw material for several purposes, such as partial substitutions of raw materials for the production of concretes and mortars [6]. This industrial sector is responsible for more than 30% of the extraction of natural resources, in addition to 25% of the solid waste generated in the world. This is because the industry generally adopts the linear economic model. Thus, a paradigm shift has been necessary in the industrial scenario with the adoption of a circular economy model [11]. Considering circular economy practices, it is possible to associate it with life cycle assessment (LCA) in this process, since the tool helps in identifying the benefits of reusing materials and reducing the amount of natural resources [11]. LCA classifies environmental impacts through a lifecycle perspective, providing valuable information that determines better strategies for decision making [12].

**Figure 1:**
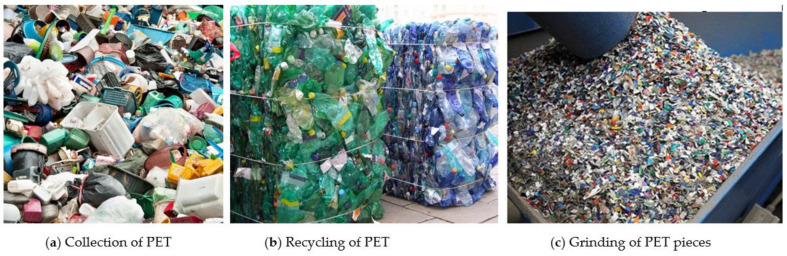
Stages for application in building materials.

According to the United Nations (UN) for the Environment (2019), the world produces about 300 million tons of plastic waste annually, but only 9% of the plastic waste generated is recycled and 14% is collected for recycling. In the oceans, 90% of the plastics generated on the planet are deposited. Plastics are used in large quantities due to their beneficial properties, such as lightness, high strength, flexibility in transforming into different shapes, and high resistance to bacteria. However, as it is widely consumed, it ends up generating large amounts of waste. Most plastics produced are suitable for short-term applications, while about a quarter is used for long-term applications, such as piping. Short-term use has triggered an increase in plastic waste annually and, consequently, inappropriate disposal in the environment [13] as well as the cullet waste [14]. Figure 1 shows PET waste in the collection (a), recycling (b), and grinding (c) stages for application in building materials [15,16].

From an environmental point of view, a separate collection and recycling system for post-consumer discards can contribute to improving environmental degradation and economic benefits [17,18]. Post-consumer plastic is quite outdated due to the high level of contaminants, odors, and substances added intentionally. Currently, innovative decontamination is a necessary technology to prepare recycled material for applications that require this type of care. For this to be possible, the upcycling process must be implemented, providing that the material is prepared to be properly reused [19]. According to the report “The State of Plastics” carried out by the UN Environment (2018), the most found discarded plastics are cigarette butts, PET bottles, PET bottle caps, food wrappers, and bags. Although PET is a recyclable material, most are disposed of in inappropriate places, causing huge losses. This is related to the total cost of the lifecycle, corporate responsibility, as well as the lack of dissemination of public information and the lack of incentives for recycling cooperatives [20,21].

Considering these issues, several studies [22,23,24,25] have been carried out, showing an alternative for the destination of PET waste as an addition in alternative construction materials, mainly for applications that do not require large deposited and structural loads. 

In this context, the objective of this work was to carry out bibliographic research regarding the reuse of plastic waste, mainly polyethylene terephthalate (PET), in different building materials. The methodology used was to search for the information present in this review through literature and articles in the academic databases: Scopus, Science Direct, and Web of Science, using as selection criteria: circular economy, sustainable development, lifecycle assessment, circular economy, environmental impact, plastic waste, PET waste, construction materials, and civil engineering. The main innovations of this article are to carry out LCA applied to plastic waste in building materials. Although there are other reviews on plastic waste applications, these manuscripts do not address the proposed theme based on LCA concepts. This can help further research on the response to the application of these wastes in the long term, and not only on immediate responses, proving the true importance of recycling plastic in construction materials, especially regarding its more efficient application.

## 2. Circular Economy

According to Krishna et al. [6], the generation of wastes should be considered as an opportunity for the development of the industrial sector, since it can add value to the wastes generated, encouraging the circular economy that directly influences the growth of the sector and society. Currently, the economy is geared towards the linear model, which extracts natural resources, transforms, consumes, and discards. In the circular model, on the other hand, everything that is extracted, somehow, goes back into the cycle [26]. Material recovery is considered one of the central principles of circular economy. Thus, the better the reuse of waste, the closer to its strategic objectives the production will be and, consequently, its profit will be greater [27]. Such an approach is increasingly seen in building materials engineering. Examples include the use of fly ash in concrete production [28]. Fly ash as a by-product of coal combustion in power plants is a raw material that is extremely dangerous for the environment, so often, attempts to manage this waste are inscribed in the key environmental problems of countries based on the coal power industry. The utilization of this waste in building materials is compatible with sustainable development and promotes the improvement of environmental conditions [29]. Another example is the already widespread use of waste aggregates, e.g., ceramic [30,31] or glass [32,33], as a partial or total substitute for natural aggregates. Such approaches mean that the composite obtained very often not only completely balances the effect of natural aggregate but can also add new values to the material. For example, a cement composite with ceramic waste is much more resistant to the effects of elevated temperatures than its counterpart made of natural aggregates [30]. In the following part of the paper, it is shown that the use of PET as a secondary raw material in the production of construction materials is also fully in line with the sustainable trend.

The circular economy assists in the preservation of limited stocks of raw materials, in addition to reducing greenhouse gases. Such an approach defends the preservation of active materials, contrasting with the culture of consumption continuously. Within the key sustainability guidelines in the treatment of natural resources as a valuable set of resources is to produce with less degradation, being contrary to the linear economy [34]. In this type of economy, the objective is to increase the life stages of each product through a continuous development cycle. This process preserves and supports the durability of natural capital. In addition, it is important to highlight the complexity involved in creating these mechanisms, which involves innovation research, and efforts by economists and environmentalists [34]. In addition, product design plays a significant role in promoting the circular economy. The flow of consumables must be kept clean so that the materials can be recycled more efficiently.

**Figure 2:**
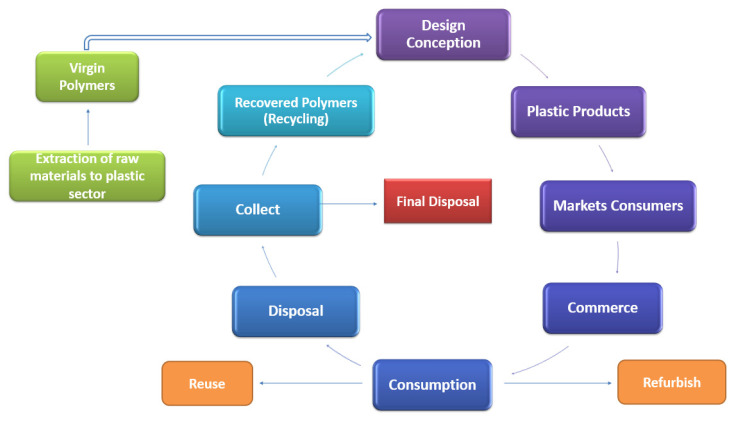
Circular system of plastic products.

Along with this, the stock in use must be minimized, avoiding the stagnation of resources. The collection and cost of primary material must be facilitated so that the price of secondary material, which is recycled, is kept lower than that of the primary material, which is virgin [35]. The flowchart shows the circular system of plastic products (Figure 2).

## 3. Sustainable Development

The sustainability involved in waste management provides a better decision-making process and supportive policies, in addition to appropriate treatment technologies, ideal waste for reuse, and methods of resource recovery, reaching the objectives of sustainable development [36].

**Figure 3:**
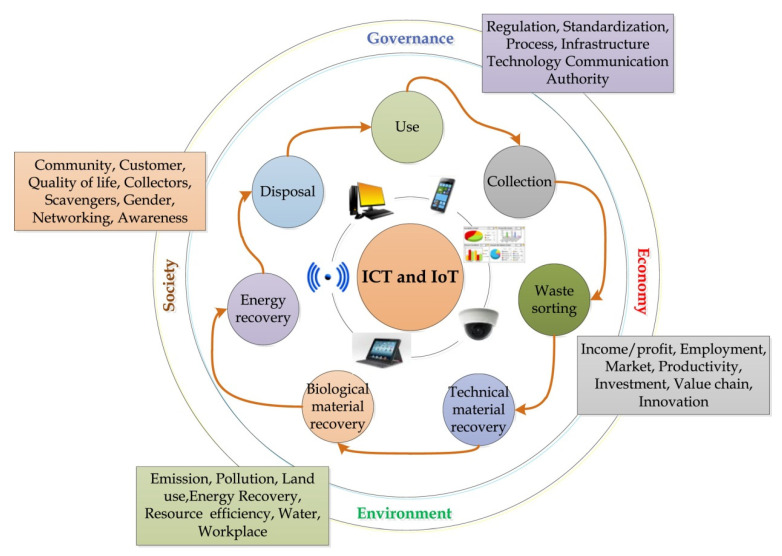
Framework of a sustainable and smart waste management system [36].

One of the main requirements for sustainable development is to ensure greater protection of the environment and natural resources, providing greater capacity to meet the demands of future generations, in addition to our current needs. Along with this, it is necessary to develop strategies with urban, industrial, agricultural, logistics, and technological policies to establish the basis for sustainable development [37]. Fatimah et al. [36] showed the fundamental framework of a sustainable and smart waste management system through a flowchart (Figure 3).

Such a need and the excessive extraction of mineral resources in civil construction have caused the sector to adapt its processes, including sustainable construction materials [37]. With this in mind, the concept of circular economy can be associated due to the concerns regarding the significant consumption of resources in the construction industry. In this way, a successful transition to sustainable development in the construction industry can be promoted [38].

The main challenges for the management of solid waste are associated with the main aspects of the waste sector, which are the generation and processes of collection, transportation, treatment, and final disposal [5]. Integrated management of solid waste using different methods, such as incineration, composting, anaerobic digestion, fuel derived from refuse, land reclamation, and dumps, is very necessary [39]. Currently, the construction industry seeks to improve social, economic, and environmental indicators regarding sustainability [40]. These issues can be linked through the LCA, which provides a large number of possibilities for data entry, within a given lifecycle scenario that you want to be analyzed, in addition to providing the critical points of the process that need to be improved [41].

## 4. Lifecycle Assessment (LCA)

There are currently different approaches to and perspectives of measuring the impact of a specific product on a system in LCA [19,42]. Along with the changes in the current economic scenarios, regarding the development of alternative materials, greater attention is not only focused on the problem of waste reuse, but also on the environmental management tools used in the process. LCA has been highlighted when analyzing alternatives that have sustainable potential for a given process, developing improvements for the environment and for the consumer [43].

**Figure 4:**
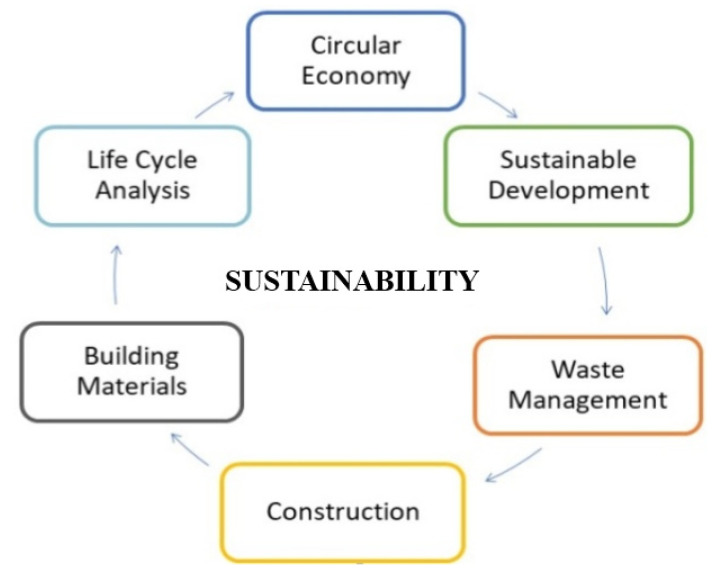
Relationship between lifecycle assessment and sustainability in civil engineering.

The application of LCA is carried out in a sequence of pre-defined steps, according to the standardization process (ISO 14040), which in addition to products, encompasses processes or services that quantify and characterize the input and output statistical data of matter and energy, in addition to classifying it into impact categories. It makes it possible to understand the action of a product system on the environment, which makes the tool an interactive process [44]. Among the various environmental management techniques, such as risk assessment, environmental auditing, and environmental impact assessment, LCA acts as a simplified modeling of reality, with the greatest challenge being the development of a model in which process simplifications and distortions do not cause a significant influence on the expected results [43]. In Figure 4, it is possible to verify the interrelationship between LCA and sustainability in the civil construction sector.

## 5. Plastic Wastes Applied in Civil Engineering

**Figure 5:**
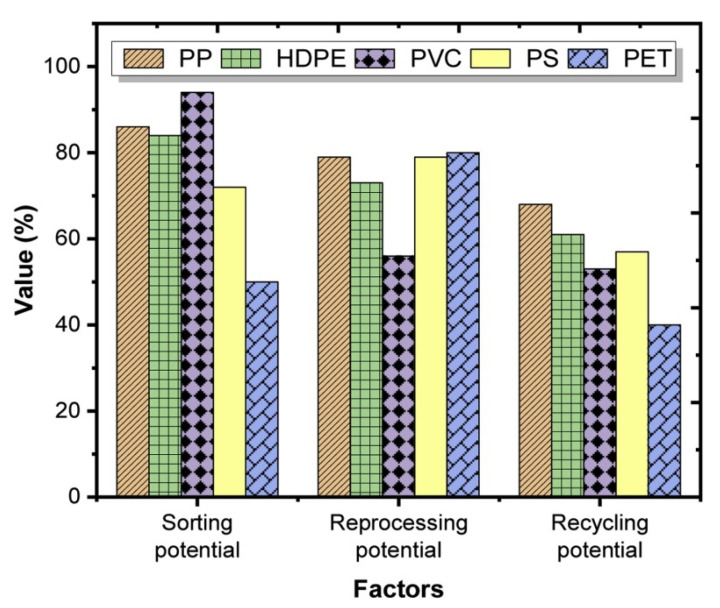
Recycling potential of the various types of plastics [13].

Several types of plastics can be applied in the construction industry and have recycling potential as shown in Figure 5. A study by Awoyera and Adesina [13] indicated that depending on the type and properties of the plastic waste, they may have potential use in various applications after recycling. Thus, high-density polyethylene (HDPE) is a relatively hard and rigid material and can find application in the manufacture of plastic lumber, tables, chairs, and other furniture. The light-density polyethylene (LDPE) is a flexible material and can potentially be used in the production of bricks and blocks. Polypropylene (PP) is hard and flexible, and due to these properties, its potential applications are aggregates in asphalt mixtures. Polystyrene (PS) is hard and brittle, so it is mainly used for parts that are not highly stressed mechanically, e.g., insulation materials. Polyvinyl chloride (PVC) is hard and rigid, which indicates its potential use as an aggregate in cement-based materials. PET, on the other hand, is hard and flexible and the most common secondary application is as fibers in cementitious composites.

In this work, the polyethylene terephthalate (PET) polymer is highlighted. It is possible to verify the incorporation of PET in different types of construction materials, as shown in Table 1.

After analyzing Table 1, it is noted that in the construction industry, plastic waste can be beneficially used for various purposes, for example, as aggregate in cement-asphalt mixtures or insulating materials. Despite the great potential, the use of plastic waste in construction is still limited. The use of plastic waste in cement composites paves the way for reducing the environmental burden from the extraction of natural aggregates. In terms of mechanical properties, the use of plastic waste as fiber in cement composites does not adversely affect compressive strength. It has also been found that using recycled plastic as fibers in cement composites controls plastic shrinkage. A significant improvement in the thermal properties of cement-based materials can be achieved through the addition of recycled plastics. The ability of plastic waste to improve the thermal properties of a cement-based material can be attributed to its low thermal conductivity. Research has shown that the use of plastic as an aggregate in subfloor and sub-pavement construction improves the shear resistance, stiffness, and load-bearing capacity of the pavement. However, it was concluded that 5% is the optimal amount of plastic waste that can be added to asphalt mixes without any negative impact on their viscosity.

**Figure 6:**
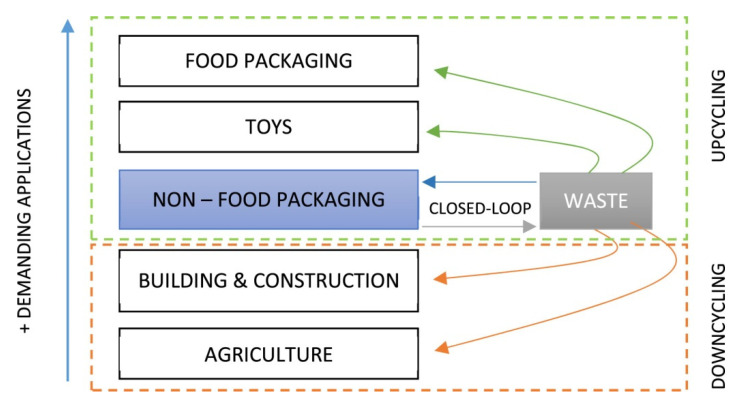
Recycling options for post-industrial plastic waste [19].

For post-industrial plastic waste, there are several possibilities for reuse and recycling, as shown in the process upcycling and downcycling diagram of [19] in Figure 6.

From the studies cited (Table 1) that use PET waste, it is possible to relate the influence of the PET percentage variation and other wastes in the mixtures to the manufacture of construction materials, such as paving, mortars, stabilized blocks, and concrete. It has a significant role together with the total composition of the material in obtaining satisfactory results in their respective technological tests, mainly with regard to its mechanical performance, as shown in Table 2.

### 5.1. Benefits of Using PET Wastes in Construction Materials

The studies presented above point to a number of advantages of using PET wastes in the production of mainly cement-based materials. The most important ones include:

- Reducing the costs associated with the waste management. The plastic waste management helps reduce the amount of plastic wastes in dumps, which reduces storage costs.
- Lower price of construction materials. Since PET is a waste and its storage is costly, its recycling and reuse makes it very cheap in comparison with classically used materials. The financial benefits are multifaceted, i.e., very low price of the raw material for the recycling plant, very low price of the processed raw material for the construction material manufacturing plant, and lower dump maintenance cost for the plastic waste recycling company.
- Development programs in many countries. Many countries with a high level of plastic waste pollution and many others have development and implementation programs to adapt the plastic raw material for industrial reuse. Thus, there are additional funds to support such activities, and the entrepreneur interested in the waste material may obtain a grant from the state for this purpose.
- Improving the properties of materials made with recycled PET. Cement-based materials made with PET are characterized by no worse strength parameters in comparison with classical cement composites. Their absorbability is mostly lower because PET waste itself has very low absorbability. Increased resistance to bio-corrosion characterizes construction materials made of PET.
- Reducing the energy demand of a building. The use of plastic wastes in the production of insulation materials improves the energy performance of buildings, which reduces the cost of its maintenance.
- Low transportation costs of PET wastes. This type of waste is locally available almost everywhere. Their use reduces the costs of transportation of waste to the target processing plant, compared to natural raw materials, whose excavation sites can sometimes be far away from the place of their application. This approach also reduces CO_2_ emissions and other pollutants associated with transport.

### 5.2. Risks and Limitations Associated with the Use of PET Wastes

Based on the literature review, a number of risks and limitations that are associated with the potential use of PET wastes were also detailed. The most important of these are listed below:

- Sometimes high contamination with different compositions. PET wastes are very often contaminated with other materials, which are often specks at a further processing stage. In such cases, it requires the application of additional treatment to remove the speck components.
- The public is unaware of the harmlessness of PET wastes embedded in construction materials. PET wastes enclosed in the matrix of construction materials are totally harmless to the environment and to the users of the facility built with such materials. Plastic wastes are still perceived as highly hazardous waste; however, its reuse usually reduces its harmfulness. Unfortunately, the public is still very unaware of this issue.
- Lack of standards and regulations for the use of PET wastes in the manufacturing technology of construction materials. At present, a vast majority of PET wastes applications are based on the results of scientific research and development works. There are still no standards for their processing and methods of incorporation into the structure of construction materials, which limits their commercial application.
- Poor interface of PET wastes with the matrix of construction material. This is one of the main problems associated with PET application in the civil engineering, but it is also true for other plastic waste. This is mainly due to the low surface energy of the PET wastes, which can sometimes result in compromised mechanical cohesion of the finished composite.
- Lack of knowledge regarding the long-term performance of building materials with PET wastes. As this is a relatively recent trend in building materials engineering, there is still a lack of information regarding the long-term durability of recycled construction materials. Thus, there are sometimes concerns from contractors about the commercial use of wastes for this purpose.
- Variability in composition. PET products are made in a variety of grades, species, and types, which sometimes results in different properties between the different assortments. This requires the development of ways to segregate PET wastes according to their properties. This will allow for the proper design and control of the properties of PET construction materials.
- The generally low density of PET wastes. In some construction applications, low density is desirable, e.g., insulation materials or lightweight structural concretes. This often limits their use in cases where high stiffness and strength is required.

## 6. Sustainability in Civil Engineering through the Lifecycle Assessment (LCA) Using PET

**Figure 7:**
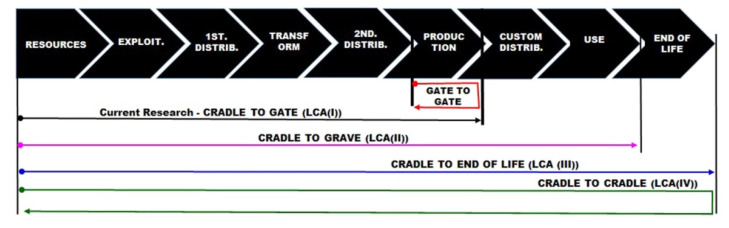
LCA boundary conditions [61].

It is possible to adopt or point to a specific result in performing LCA instead of using only a single parameter approach or consensus. The impact categories are generally correlated and, therefore, a single choice between them can be quite significant for the entire assessment [60]. Figure 7 represents a scheme, based on ISO 14040, where it is possible to verify the conditions to establish the boundary in LCA [61]. 

Using Table 3 and Table 4 it is possible to verify the use of LCA in different works with regard to the application in civil engineering and recycled plastics. Thus, the tool allows studies focused on the theme to be approached in a more holistic and sustainable way. It was possible to list different products, categories, and approaches analyzed by the authors.

**Figure 8:**
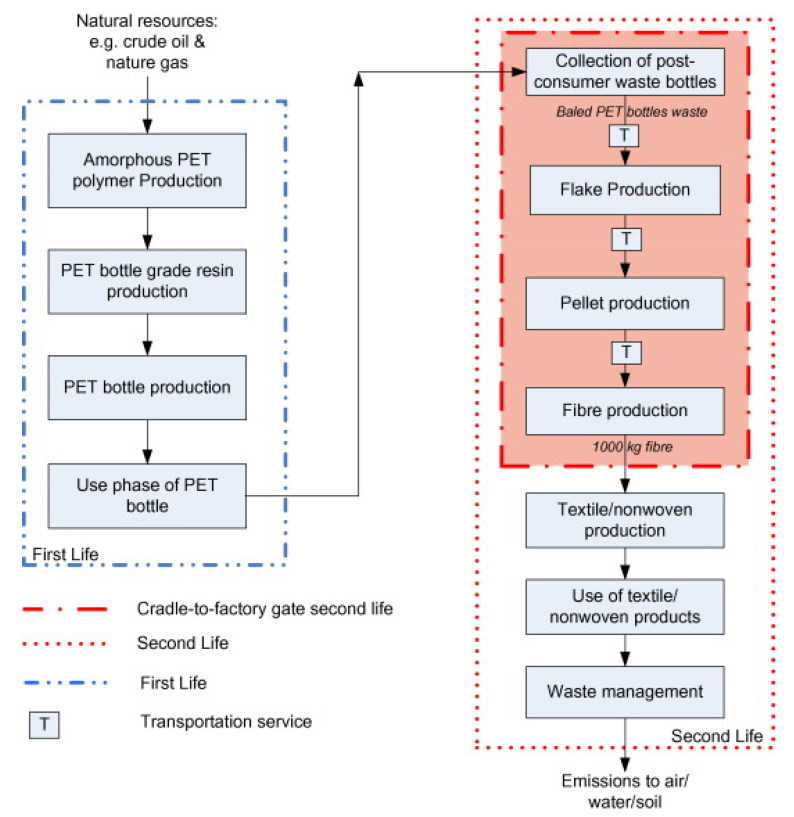
Flowchart of PET waste recycling [71,75,76,77].

**Figure 9:**
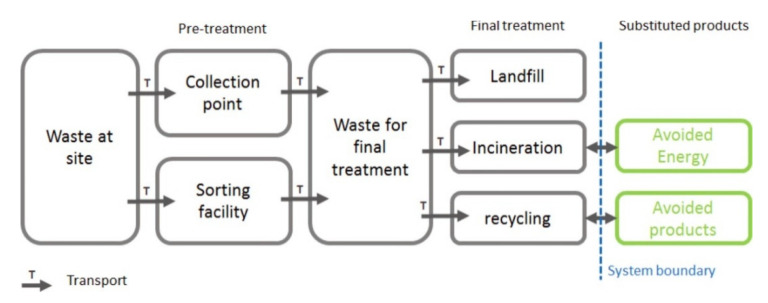
Flowchart of the boundary system [65].

**Figure 10:**
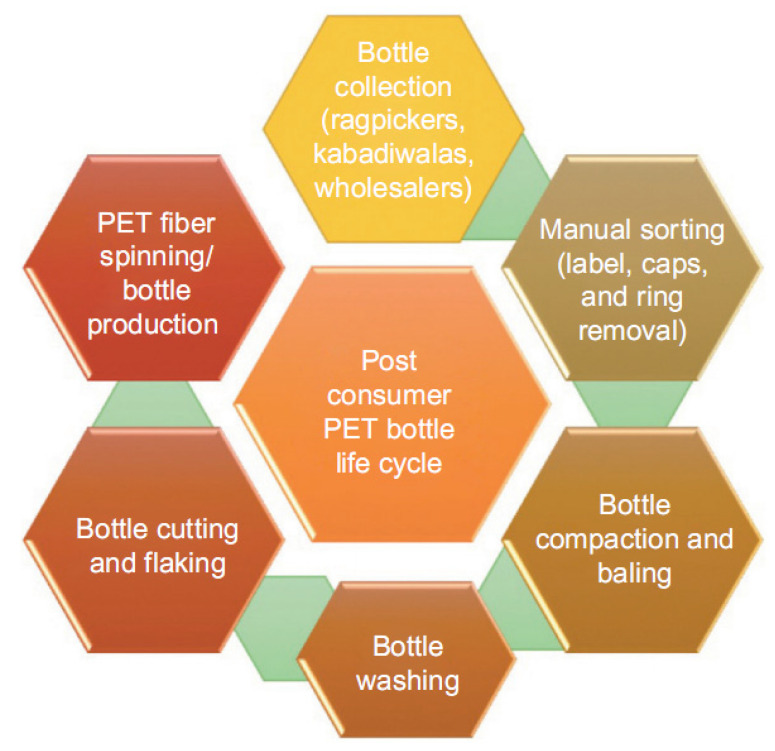
Flowchart of the LCA system [66].

The related literature on this topic also shows the flexibility and importance of choosing scenarios for the respective studies and border systems. Most studies clearly define the objective and scope of the study, making it easier for readers to understand the different criteria considered in each of them, such as [63,64,65,66,67,68,69,70,71,72] (Figure 8), [65,66,67,68,69,70,71,72,73,74] (Figure 9), and [66,67,68] (Figure 10).

Figure 8 shows a PET waste recycling system, including bottle shredding, smelting, and new product creation. At the same time, electricity must be used at all stages [63,64,65,66,67,68,69,70,71,72].

In civil engineering, the lifecycle assessment in elements, such as walls, with different end-of-life potentials, is mainly related to direct reuse residues or demolition processes, which depends on the users. Several models can be proposed for this purpose, and research was carried out in order to categorize them into five different scenarios, which followed the guidelines of the environmental profile of building elements (EPBE). Figure 9 presents three pathways for PET waste, such as dump, incineration, and recycling. Obviously, the first two paths are environmentally dirty. If plastic waste is dumped, soil contamination can occur. If plastic waste is incinerated, the atmosphere will be polluted with harmful substances in large quantities. 

Figure 10 shows the lifecycle of plastic bottles after use [66,67,68]. First, they need to be collected in a separate garbage container, requiring consumers to take responsibility for this step. All non-plastic items, such as labels, caps, etc., are then removed from these bottles. After this, the bottles are compressed and washed. As such, they can be cut into thin strips and/or used for recycling bottles.

## 7. Conclusions

From the literature cited in this paper, it is possible to state that the implementation of LCA provides numerous alternatives to enhance detailed studies regarding environmental and economic impact assessments. As a result, there is also a greater appreciation of the product and the process considered.

Parallel to this, it was possible to verify that the work regarding the reuse of plastic waste in the construction sector mainly concentrates the justifications around the issue of alternative recycling for this waste, but this is no less important. However, more holistic studies are needed regarding plastic waste incorporated in the construction sector, as this can promote a long-term perspective, making it possible to consider more complete and long-term studies.

For future work, economic evaluations can be considered about recycled plastics, which can measure the effects of the savings generated by the incorporation of PET, in addition to the environmental advantages for the construction sector. In addition, detailed studies are also necessary that involve the issue of loss of the economic value of the waste due to the amount of time that the material is disposed of without applications, and recycling steps can be included in the life cycle analyses, since these are hardly considered.

Regarding the use of PET waste in the construction sector, the study allowed us to verify that several factors contribute to its use, such as, for example, the percentage variation in different construction materials. It was possible to affirm that the adequate amount of addition of this waste mainly influences the mechanical performance of the manufactured material and, therefore, attention must be given to this stage of the manufacturing process, together with the pre-treatment of the recycled waste, including the particle size of the waste aggregate.

Through this article it was possible to prove the main alternatives studied by the authors for the destination of plastic waste, such as ethylene polyethylene, in the construction sector. Along with this, it was possible to report that LCA provides greater quantification of information, making the alternative process or product more valued, especially regarding the environmental impact assessments.

It was possible to conclude that studies focused on approaches are still lacking considering the most complete cycle, which provides a more holistic and interesting view of the incorporation of plastic waste in the construction sector, as well as in terms of circular economy, that is, being able to add value to waste. Thus, studies are not limited to justifications for the destination of these residues.

## Figures and Tables

**Table 1:** Works that incorporate PET in construction materials found in the academic databases.

Title	Year	Type of Material	Studied Residue	Refs
Influence of PET wastes on the environment and high strength concrete properties exposed to high temperatures	2019	Concrete	Crushed PET of drinking water bottles with fly ash	[23]
Utilizing recycled PET blends with demolition wastes as construction materials	2019	Paving	Crushed PET bottles and food packaging with construction and demolition waste (concrete and crushed brick)	[22]
Evaluation of compressive strength and water absorption of soil-cement bricks manufactured with addition of pet (polyethylene terephthalate) wastes	2016	Soil-cement block	Crushed PET of drinking water bottles	[45]
Experimental behavior and analysis of high strength concrete beams reinforced with PET waste fiber	2020	Concrete	Crushed PET of drinking water bottles	[25]
Study on behavior of concrete with partial replacement of fine aggregate with waste plastics	2019	Concrete	Milk pouches, Polythene bags, water bottles	[14]
Analysis of physical and mechanical properties of pressed concrete blocks without structural purposes with additions of recycled PET	2019	Concrete	PET recycled and crushed from a recycler	[46]
Stiffness and flexural strength evaluation of cement stabilized PET blends with demolition wastes	2020	Paving	PET recycled and crushed from a recycler	[47]
Fracture and mechanical properties of asphalt mixtures containing granular polyethylene terephthalate (PET)	2020	Paving	PET granules	[48]
Lightweight PET based composite aggregates in Portland cement materials—microstructure and physicochemical performance	2020	Mortar	Flakes from recycled packages PET	[49]
The selected mechanical properties of epoxy mortar containing PET waste	2015	Mortar	Glycolisates of PET waste from two chemical plants	[50]
Study of the suitability of unfired clay bricks with polymeric HDPE & PET wastes additives as a construction material	2020	Unfired clay brick	Grinded PET flakes in three sizes: δ ≤ 1 mm; 1 mm < δ ≤ 3 mm; 3 mm < δ ≤ 6 mm	[51]
Tensile performance of sustainable Strain-Hardening Cementitious Composites with hybrid PVA and recycled PET fibers	2018	Mortar	Surface treated (NaOH solution; silane coupling agent) PET fibers	[52]
Concrete incorporated with optimum percentages of recycled polyethylene terephthalate (PET) bottle fiber	2018	Concrete	PET bottle fibers (50 mm length, 5 mm width)	[53]
Recycling woven plastic sack waste and PET bottle waste as fiber in recycled aggregate concrete: An experimental study	2018	Concrete	PET bottle fibers (50–60 mm length, 2–3.5 mm width)	[54]
Durability performance of a novel ultra-high-performance PET green concrete (UHPPGC)	2019	Concrete	PET fibers (40 mm length, 3.5 mm width) obtained by using a simple shredded machine	[55]
Evaluation of Material Modification using PET in 3D Concrete Printing Technology	2021	Mortar	PET granules (2–5 mm size)	[56]
Performance of mortars with PET	2021	Mortar	Grounded PET wastes	[57]
Post-fire compressive strength of recycled PET aggregate concrete reinforced with steel fibers: Optimization and prediction via RSM and GEP	2020	Concrete	PET chips	[58]
Strength and toughness characteristics of AC-WC mixture containing PET and PP plastic waste under static compression	2021	Asphalt-concrete mixture	Shredded PET bottles	[59]

**Table 2:** Relationship between the percentage of PET incorporation and other residues in the composition of construction materials with the mechanical performance of the materials.

Refs.	Percentage of Waste Used	Main Results Found by the Authors
[22]	3% and 5% (PET with concrete and brick waste)	The mixtures of PET with concrete aggregate and crushed brick performed satisfactorily and the mixtures considered satisfactory for all the analyzed requirements (particle size distribution, particle density, sieve analysis, flaking index, Los Angeles abrasion, absorption of water, Proctor compaction, hydraulic conductivity and California support index) for application on pavement sub-bases.
[23]	0.25% PET; 30, 35%, 40% fly ash; 2.5%, 5%, 7.5% nanosilica material	Compressive and flexural strengths were improved by the presence of fly ash and nanosilica material, but it was reduced by controlled temperatures. Splinters appeared in samples containing nanosilica material exposed to high temperature, but not in samples containing PET combined with nanosilica. The porosity of the control samples increased with increasing temperature, while the presence of fly ash and nanosilica in concrete samples refined the pores by 50%. The color of the surfaces changed from dark at room temperature to light with increasing temperature. Samples containing PET waste and exposed to high temperatures release a greater amount of CO (carbon monoxide).
[45]	20%, 15% and 10% PET	Soil-cement blocks with 10% PET added reused around 300 g of PET in each block, although the results showed low values of compressive strength, but still representing an alternative solution for masonry works without large loads, with satisfactory water absorption.
[25]	0,75% and 1% PET	Maximum loss of compressive strength of about 30% with the use of long PET fibers (40 mm), as opposed to the use of short fibers (20 mm) in concrete, which took a relatively small loss. The presence of PET fibers (mainly in the dosage of 0.75% by volume) in high-strength concrete has a beneficial effect to control pre-cracking deformation, especially regarding the crack control capacity in the elastic band.
[14]	15%, 20%, 30%, 40% and 50% PET	The greater the addition of added plastic waste, the lower the compressive strength of the concrete. The authors stated that this type of material can be used for the construction of temporary structures, with lower applied loads, such as sealing structures and even lining of river channels, since there is less chance of corrosion due to the presence of plastic.
[46]	15%, 30% and 45% PET	The pressed concrete blocks that showed the best behavior were those with 15% PET, since their resistance to compression showed a higher value than the others, highlighting even less absorption due to the greater degree of packaging and better homogeneity of the mixture.
[47]	3% and 5% PET	The addition of PET decreased the values of the resilience module of construction and demolition (C&D) waste, while with crushed bricks they presented higher values of resilience module than with recycled concrete. The PET mixtures with crushed bricks showed a higher flexural and fatigue modulus than PET mixtures with recycled concrete. The mixtures stabilized with cement containing 5% PET with C&D residues presented physical and mechanical properties that meet the technical requirements for the construction of the base and sub-base of pavement.
[48]	0%, 30%, 50%, 70% and 100%	The resistance was reduced due to the PET presence, but the moisture resistance of the mixtures produced slightly improved. The gradual increase in PET content increased the rigidity of asphalt mixtures and provided an increase in fracture energy, especially when the PET content adopted was 70%. The fracture resistance was reduced when 30% and 50% PET were added. The probability of fracture failure increased significantly as the test temperature was reduced. They concluded that with the increase of PET in the mixtures, the susceptibility to fracture failure increased.
[49]	10%, 25% PET and fly ash	Mixing with PET using both types of fly ash improves the usable properties of the mortars obtained compared to the mortar with PET alone. Regarding the modeled mechanical properties, they observed an increase in the resistance to compression or flexion due to the possibility of reducing the water/cement ratio in the mixtures without loss of workability.
[50]	0–14% Glycolisates PET	The application of plastic waste allows the production of polymeric mortars of very good resistance, hardness, mainly with the substitution of an amount of epoxy of 9%, in weight, besides reducing even the production costs.
[51]	0%, 1%, 3%, 7%, 15%, and 20% PET flakes and HDPE	Increase in porosity of unfired clay brick with increase in PET content. Decrease in density below 1.75 g/cm^3^. Increased capillary rise capacity with increasing PET content and fraction. Decrease in compressive strength.
[52]	Substitution of the PVA fibers by PET fibers up to 50%	Strain hardening mortar with PET fibers achieved robust tensile strain-hardening and multiple cracking for general applications. With an increased content of PET fibers, the uniaxial tensile performance deteriorates to 3.63 MPa. The use of PET fibers greatly reduced the material cost and environmental impact.
[53]	0.5%, 1.0%, 1.5%, 2.0% of PET fibers	The slump test value and compressive strength decreased with increasing fiber content. Tensile strength increased.
[54]	0.25%, 0.50%, 0.75% of PET fibers; the same content of recycled woven plastic sack	The PET fibers were applied in the recycled aggregate concrete. The fibers have high alkali resistance in alkaline environments. The decrease in tensile strength after storing the composite for 180 days in an alkaline solution was negligible (0.07–0.6%). The combined use of fly ash and PET fibers resulted in an increase in compressive strength in the range of 3.6–9%. However, as the fiber content increased, the strength decreased. The modulus of elasticity increased in the range of 16.9–21.5%, the Poisson’s ratio increased in the range of 4–8%. The tensile strength of concrete with PET fibers was higher by 11.8–20.3%.
[55]	1% of PET fibers; 20% and 40% of ultrafine palm oil fuel ash	Reduction in slump and viscosity was observed after addition of the PET fibers. The combination of ultrafine palm oil fuel ash and PET fibers resulted in an increase in compressive strength at all curing times i.e., after 3, 7, 14 and 28 days. After 28 days a very high strength of 144.1 MPa was achieved. The combination of the two materials also improved the bonding properties between the PET fibers and the cement matrix.
[56]	30% of PET granules	The suitability of PET granules was evaluated for use in the mortar designed for the 3D printing technology. PET granules were not found to adversely affect the rheological properties of the fresh mix. A 30% decrease in compressive strength was observed for standard samples and a 10% decrease in strength for printed ones.
[57]	5 and 10 % of grounded PET	The grounded PET was used as a replacement for aggregate in mortar. A decrease in mechanical strength was observed. The beneficial change was an increase in resistance to capillary rise of water and a decrease in thermal conductivity.
[58]	5 and 10% of PET chips	PET chips were used as a replacement for sand. A decrease in compressive strength was observed over the entire range of temperatures analyzed, i.e., after 25, 200, 400, and 600°C. At 600°C, the decrease in strength relative to 25°C was 60.9% for samples without PET chips and 82% for samples with PET chips.
[59]	3% of plastic wastes—PET:PP in a ratio 100:0, 0:100, 50:50	An increase in compressive strength of asphalt-concrete mix with PET was observed. However, there was no significant difference in Poisson’s ratio and toughness index values.

**Table 3:** Works that used LCA for environment and economic impacts assessment.

Title	Year	Refs.	Product Analyzed
Environmental and economic impacts assessment of concrete pavement brick and permeable brick production process—A case study in China	2018	[62]	Traditional and permeable concrete blocks
Environment and economic impacts assessment of PET waste recycling with conventional and renewable sources of energy	2019	[63]	Use of renewable (solar) energy for PET recycling processes
Environmental life cycle assessment of traditional bricks in western Maharashtra, India	2014	[64]	Pollutant emissions in the manufacture of traditional clay bricks
Challenges in life cycle assessment clay-based construction materials clay-based construction materials	2017	[61]	Environmental impacts of clay-based and clay-free building materials effects of clay-based and clay-free building materials
Sustainability assessment of circular building alternatives: Consequential LCA and LCC for internal wall assemblies as a case study in a Belgian context	2019	[65]	Quantified assessment of the potential environmental and financial benefits and burdens of introducing circular design alternatives for internal wall assemblies to the Belgian market/ Reviews the methodological implications on the results of a consequential LCA and a life cycle costing (LCC), acknowledging the time dependence and closed-loop nature of those circular design alternatives
Life Cycle Assessment (LCA) of PET bottles	2019	[66]	Environmental impact associated with different stages of the PET bottle life cycle such as production, transportation, and recycling
Recycling in buildings: an LCA case study of a thermal insulation panel made of polyester fiber, recycled from post-consumer PET bottles	2011	[67]	Eco-profile, energy savings and the environmental benefits of the use of PET recycled bottles for the manufacture of thermal insulation
Life-cycle assessment (LCA) aspects and strength characteristics of self-compacting mortars (SCMs) incorporating fly ash and waste glass PET	2019	[68]	Self-compacting mortars with 80% of Portland cement, 20% fly ash, and 3%, 6%, and 9% of glass PET waste as a substitution of fly ash
Comparative Life Cycle Assessment of Incorporating Recycled PET Aggregates into Concrete.	2019	[69]	Concrete with the 0%, 14%, 47%, and 58% of recycled PET aggregate as fine aggregate

**Table 4:** Summary of categories and perspectives used in the LCA studies.

Authors	Main Impact Categories Analyzed	Approach Used
[62]	Raw material inputs, energy consumption, transport, waste and wastewater discharge, permeability, water, sand, cement, diesel, electricity	cradle-to-gate
[63]	Ecosystem quality, health, human resources, climate change, fossil depletion, human toxicity, ozone depletion, terrestrial acidification, and water depletion	cradle-to-grave grave to grave
[61]	Global warming (emission of CO_2_, CO and CH_4_), acidification, eutrophication (emission of SO_2_, NH_3_ and NOx) and depletion of the ozone layer	cradle-to-gate
[64]	Carcinogens, resp. organic, resp. inorganic, climate change, radiation, ozone layer, ecotoxicity, acidification, land use and minerals	grave-to-grave
[65]	Onsite sorting, transport, pre-treatment, distribution between landfill, incineration, and recycling	grave-to-grave
[70]	Acidification, carcinogens, eutrophication, ecotoxicity and exaggeration	cradle-to-grave
[67,71,72,73,74]	Global warming potential, ozone layer depletion, photochemical oxidation, acidification, eutrophication	cradle-to-gate
[68]	Climate change, ozone depletion, terrestrial acidification, freshwater eutrophication, marine eutrophication, human toxicity	cradle-to-gate
[69]	Climate change, ozone depletion, human toxicity—cancer effects, human toxicity—non-cancer effects, particulate matter, acidification, terrestrial eutrophication, land use, water resource	cradle-to-gate

## Data Availability

Not applicable.
